# Burnout among Telecommunication Sales Managers

**DOI:** 10.3390/ijerph191811249

**Published:** 2022-09-07

**Authors:** Romualdas Malinauskas, Mantas Grinevicius, Vilija Malinauskiene

**Affiliations:** Department of Physical and Social Education, Lithuanian Sports University, Sporto 6, LT-44221 Kaunas, Lithuania

**Keywords:** sales managers, burnout dimensions, emotional exhaustion, depersonalization, personal accomplishment, workplace bullying, job stress

## Abstract

(1) Background: Various investigations have confirmed that burnout prevails in intensive and demanding contemporary working environments. Most of these studies have analyzed the associations between emotional exhaustion and various work factors. We studied the gap in the literature by simultaneously considering the three commonly recognized dimensions of burnout (emotional exhaustion, depersonalization, and reduced personal accomplishment) using a representative sample of telecommunication sales managers. (2) Methods: 849 survey respondents completed an anonymous questionnaire that included items representing psychosocial factors at work, lifestyle characteristics, and the Maslach Burnout inventory. The hierarchical regression analysis revealed the predictors of emotional exhaustion, depersonalization, and reduced personal accomplishment. (3) Results: job demands and witnessing bullying at the workplace were the most powerful predictors of emotional exhaustion, followed by self-rated health, night work, education, and physical inactivity. Witnessing bullying at the workplace, job control, self-rated health, and physical inactivity were the strongest predictors of depersonalization. Finally, direct experiences of negative acts at the workplace, job control, social support at work, bullying exposure duration, family crises, physical inactivity, smoking and alcohol, and body mass index were the most important predictors of reduced personal accomplishments. (4) Conclusions: the present study fills a gap in the research surrounding the three dimensions of burnout. The findings not only confirm that high job demands, low job control, and low social support at work contribute to burnout but also contribute to the novel understanding that workplace bullying plays an integral role.

## 1. Introduction

The work environments of telecommunication sales managers are always challenging, involving numerous daily face-to-face client interactions and communication with existing customers by telephone. Furthermore, these employees must keep pace with telecommunications innovation, technology, and telecommunications market trends, learn to withstand customer objections, and train junior assistants. To “win” the business, salespeople must regularly overcome stiff competition. Meanwhile, in addition to closing sales, telecommunication sales managers must often interact with angry clients who have been disappointed by a poor product and/or service. These emotionally charged interactions often engender job-induced tension [1]. Exceeding quotas, gaining new customers, and outperforming competitors and colleagues are sources of rewards, with personal income augmentation, firm revenue and profits, and customer satisfaction representing important outcome implications. Meanwhile, failing to meet assigned productivity objectives (e.g., quotas) and addressing customer complaints represent primary sources of stress [1]. Recent studies have indicated that sales managers frequently face physical, emotional, and mental stresses that may produce burnout [2]. In this context, burnout describes the expected negative impact of stress on sales managers, which manifests as reduced motivation to meet performance goals.

More generally, burnout describes a process (or syndrome) that manifests in reactions to chronic stress commonly experienced by people who provide services to other people [3]. Meanwhile, a three-component conceptualization of burnout has become widely accepted [1]. First, the emotional exhaustion component represents a response to job-related demand stressors placed upon employees (e.g., work overload and job tension). Common symptoms include dread at the prospect of returning to work, increased absenteeism, and ultimately, withdrawal from the profession. The second component, depersonalization, is a dysfunctional response to work-related stress that results from the perception that important aspects of a job are random or uncontrollable. It is characterized by a cynical or callous attitude toward some (or all) of the individual’s clients, co-workers, and superiors. Factors contributing to depersonalization include the external attribution of job-related failures and successes and a general feeling of helplessness and lack of control. The third component of burnout, reduced personal accomplishment, refers to a decline in an employee’s feelings of competence and achievement at work and stems from factors that suggest the individual is ineffective or unappreciated. Especially relevant factors include unmet achievement expectations, role ambiguity, and diminished self-efficacy [1]. Employees recognize inconsistencies between their current attitudes and their originally optimistic expectations about their careers, and they may experience a sense of inadequacy in terms of their ability to relate to people and perform their tasks [4].

Salespeople have the primary job task to bridge the gap between customers and the firm, requiring that they meet two different sets of demands, making them particularly prone to the effects of burnout [5]. Notably, studies on burnout among salespersons have often only considered one component of burnout, namely, emotional exhaustion. A recent meta-analysis study investigated six antecedents of emotional exhaustion (role ambiguity, role conflict, work overload, work–family conflict, perceived organizational support, and supervisory support), and four consequences (job satisfaction, organizational commitment, job performance, and turnover intentions). Role conflict, work overload, and work–family conflict exhibited strong positive relationships with emotional exhaustion, and supervisory support and perceived organizational support exhibited strong negative relationships [6].

Most sales research has investigated burnout without considering the sequencing of its sub-dimensions. In response to most studies focusing exclusively on emotional exhaustion [6], our research adds to the literature by investigating the associations among sales managers between the three burnout dimensions and psychosocial factors (workplace bullying and job demands, job control, and social support at work) with a concomitant evaluation of the effects of lifestyle and sociodemographic factors.

### 1.1. Psychosocial Factors at Work

Sales managers work in an environment increasingly characterized by a high degree of pressure and limited boundaries, an environment that often restricts their possibilities of self-authority and decision latitude. Sales managers must take aggressive action to train and retain sales talent, manage the sales process, and use sales support technologies to meet the challenges of this new environment [7].

The job strain theory explains how high job demands, low control, and low social support contribute to the stressful situation produced by sales managers’ working conditions [8]. High job demands and job tension can become stressors, particularly if substantial effort is required to maintain an expected level of performance [9]. When job demands are high and employees feel pressured, gaining control of the job and developing strong relationships with others helps individuals cope with stress. Low control (decision latitude) over working conditions—in terms of a lack of creativity, novelty, or the freedom and responsibility to decide what to do and when to do it—is particularly hazardous to individual health [8]. Given that social support from supervisors could attenuate the effects of burnout, it has been identified as a buffer between job-related stress and stress-related effects [10].

This questionnaire on the psychosocial factors at work also inquires about night work and physical labor (e.g., lifting, pushing, carrying, and transporting). We hypothesized that high job demands, low job control, and poor manager support negatively influence the three burnout components (i.e., emotional exhaustion, depersonalization, and reduced personal accomplishment). We also hypothesized that night work might be associated with burnout components because circadian fluctuations of the stress-related hormones—melatonin and cortisol—might be influenced by shifting work between day and night. Several macro-level job-related variables have also been included in our study, including length of stay at the company and job satisfaction. Although job satisfaction has been investigated as a burnout outcome by numerous studies, it may also act as a predictor for future burnout, given that dissatisfied persons tend to have problems overcoming burnout in the long term [11].

Bullying at work describes harassing, offending, or socially excluding an individual or negatively affecting their work, takes the form of behavior that occurs repeatedly and regularly (e.g., weekly), and lasts for a period of time (e.g., six months). Bullying is an escalating process during which the victims ultimately find themselves marginalized and the targets of systematic negative social acts [12]. Workplace bullying is a more crippling stressor for employees than all other work-related stressors combined [13]. The seriousness of the phenomenon may be supported by the fact that workplace bullying has been identified as the strongest predictor of anxiety and depression in comparison with other job-related stressors [14]. However, although bullying research is common in the context of nursing, it is very scarce for salespersons, with turnover risk [15] and work climate [16] more commonly studied. Nonetheless, one study did investigate how abusive supervision practices trigger burnout syndrome among salespeople [9]. We aimed to fill this gap by investigating bullying in the context of sales managers. We investigated 22 items representing negative acts at work, bullying exposure duration, bullying’s effect on workplace and family relations, and witnessing bullying. Notably, studies considering other occupations have found that both experiencing and witnessing bullying at the workplace may be associated with emotional exhaustion [17].

### 1.2. Internal Resources as Buffers for Stress-Induced Outcomes

Self-rated health (SRH), also known as self-evaluated health, subjective health, or perceived health, was assessed by a single question in our study. The large number of studies using this item contrasts starkly with its brevity and simplicity. Its value as a predictor of mortality and other health outcomes makes this paradox even more intriguing, especially because most studies have demonstrated an independent effect for SRH [18]. SRH has often been found to be related to external resources, including education, financial status, and social support, and internal resources, such as optimism, vigor, and perceived control [18]. Moreover, health optimism, or rating one’s health positively in response to indicators of poor ‘objective’ health, is related to lower levels of depression. Higher levels of optimism and self-efficacy help individuals manage stress better. Single items may provide unique and valuable information when they reflect the measured construct in a more psychologically meaningful way than multi-item scales [19]. Thus, we included a single question on SRH to reflect possible internal resources as a buffer for stress-induced burnout.

### 1.3. Behavioral and Sociodemographic Factors

Studies have shown that the burnout experienced by various professionals often coexists with negative emotions, which promote negative lifestyle choices. Burnout has been significantly positively associated with higher levels of fast-food consumption, more infrequent exercise, and higher alcohol consumption among health professionals from seven European countries [20]. Elsewhere, the population-based Finnish Health 2000 study indicated that emotional exhaustion relates to low levels of physical activity and heavy drinking, cynicism (depersonalization) relates to low levels of physical activity, and reduced professional accomplishment relates to low levels of physical activity, obesity, and a higher likelihood of heavy drinking [21]. The associations between burnout and adverse behavioral factors have not been considered for sales managers. Accordingly, we included physical activity, alcohol use, tobacco use, and body mass index as lifestyle indicators to investigate the complexity of psychosocial factors at work and lifestyle risk factors in hierarchal regression models to reveal their independent associations with burnout dimensions.

Finally, we included sociodemographic factors of age, gender, and education in our research, hypothesizing that these factors might be associated with burnout among sales managers based on similar studies concerning other occupational groups. For example, in a recently published systematic review and meta-analysis, burnout was substantially associated with males aged 41 to 50 with a higher level of education among physical education teachers [22]. We also investigated the length of employment at the individual’s previous company and length of service at the individual’s present company in conjunction with age, which have demonstrated influences on stress levels and burnout. Specifically, among physicians, being older and providing longer service was associated with higher levels of stress and a higher risk of burnout [23].

Notably, we also investigated family crises during the last year (unemployment, divorce, financial difficulties, and the incurable disease or death of a close family member), variables that typically represent additional stresses and may exaggerate general stress responses in everyday life, potentially contributing to burnout according to recent studies confirming the effects of stress on burnout and salesperson performance [24].

### 1.4. The Present Study

Most research studies on burnout among sales managers have investigated a limited number of burnout antecedents. Considering the multifactorial origin of burnout among sales managers, we employed hierarchical linear regression analysis to probe the associations between twenty variables—including job-related variables (high job demands, low job control, low social support at work, negative acts at work, bullying exposure duration, witnessing bullying in the previous six months, bullying deterioration of workplace and family relations, length of employment at the previous company, shift work and physical load, and job satisfaction), personal variables (family crises, self-rated health, sociodemographic: age, gender, and education), and lifestyle variables (physical activity, smoking, alcohol, and body mass index)—and three burnout dimensions (emotional exhaustion, depersonalization, and reduced personal accomplishment) in a representative sample of Lithuanian telecommunication sales managers. From a health promotion perspective, more knowledge of how various psychosocial factors, internal resources, and lifestyle habits relate to burnout among telecommunication sales managers can contribute to the attempts of occupational health professionals to organize burnout prevention implementations for this occupational category. Our study aimed to reveal the most important predictors of the three burnout dimensions in terms of effect size. Based on theoretical understandings and earlier research, we hypothesized a positive association between job demands and emotional exhaustion and hypothesized that low job control and low social support at work might predict all three burnout dimensions. We hypothesized that negative acts at work, bullying exposure duration, witnessing bullying, and bullying contributing to the deterioration of work and family relations would act as predictors of all three burnout dimensions. We hypothesized that job-related variables (night work, physical load, length of employment in the company, and job dissatisfaction), personal variables (older, females, more educated, with poor self-rated health, experienced family crises over the last year), and lifestyle variables (physical inactivity, smoking, alcohol, increased body mass index) would predict all three burnout dimensions.

## 2. Materials and Methods

### 2.1. Study Design

The study was planned as a cross-sectional survey involving telecommunication sales managers.

### 2.2. Study Participants, Procedure, and Measures

A total of 1369 telecommunication sales managers from 21 companies randomly selected from a list of 101 telecommunication companies in seven of Lithuania’s biggest cities, participated in the study. The research was conducted between October and December 2021. The questionnaire was fully completed by 849 survey respondents, indicating a response rate of 62.0%. The participating telecommunication companies passed the questionnaires to customer service and sales managers. Confidentiality and anonymity were ensured during the investigation, and no personally identifiable data were collected. Permission for the research was obtained from the heads of the divisions of telecommunications companies. Permission from the Ethics Committee of Social Sciences of the Lithuanian Sports University to conduct the research was obtained.

The anonymous and self-administered questionnaire included sociodemographic items (age, gender, education level, years in one’s last position) alongside items from previously employed questionnaires (translated and validated for use in Lithuania) designed to measure psychosocial factors, job characteristics, lifestyle factors, and occupational burnout.

### 2.3. Sociodemographic Variables

The sample comprised 37.8% (N = 321) males and 62.2% (N = 528) females. The mean age of participants was 27.78 years ± 6.69 (SD), the mean total length of service was 6.13 y ± 5.58 (SD), and the mean length of employment at an individual’s last company was 3.03 years ± 3.17 (SD). Of the total sample, 73.5% had completed university, 10.6% had completed high school, and 15.9% had completed vocational training.

### 2.4. Occupational Burnout

Occupational burnout was measured using the Maslach burnout inventory (MBI) [25,26], a 22-item questionnaire divided into three subscales: emotional exhaustion (seven items; e.g., the feeling of being emotionally overrun and exhausted by one’s work), depersonalization (seven items; e.g., the tendency to view others as objects rather than as feeling persons), and reduced personal accomplishment (eight items; e.g., the degree to which a person perceives doing well on worthwhile tasks). The items are responded to in terms of the frequency with which the respondent experiences those feelings on a seven-point scale ranging from 0 (never) to 6 (every day). The three dimensions are measured for each respondent, with a higher score indicating a higher degree of burnout, except for personal accomplishment, which is rated inversely (meaning low scores indicate high burnout). The Cronbach’s alpha in that sample was 0.925 for emotional exhaustion, 0.811 for depersonalization, and 0.797 for personal accomplishment.

### 2.5. Psychosocial Factors at Work

#### 2.5.1. Bullying in the Workplace

The 22-item negative acts questionnaire (H. Hoel and S. Einarsen) [27] was used to assess various types of negative behavior from colleagues, customers, and superiors during the previous six months (e.g., “Someone withholding information which affects your performance,” ”Being ordered to do work below your level of competence,” and ” Being humiliated or ridiculed in connection with your work”). Five answers were possible: “never,” “now and then,” “monthly,” “weekly,” and “daily.” Bullying exposure duration was also assessed (“never,” “over the last 6 months,” “over the last 7–12 months,” “3–5 years,” “more than 5 years”). In the statistical analysis, all 22 forms of negative acts were added together to provide a continuous variable for use in further analyses. Witnessing bullying during the previous six months was also evaluated (“never,” “yes, but rarely,” “yes, now and then,” and “yes, often”), as was feeling the impact of bullying in a deteriorated workplace and family relations (“never feel,” “somehow,” “a little,” and “strongly”).

#### 2.5.2. Job Demand–Control–Social Support at Work Questionnaire

Psychosocial job characteristics were measured via the job demand–control questionnaire (Theorell and Karasek), which consists of 17 items across 3 dimensions: psychological demands, job control, and social support at work [8,28]. The questionnaire includes six items to assess job control (e.g., “Can you choose HOW to work for you?“ and “Does your job require initiative?“), five items for psychological demands (e.g., “Does your job require a lot of effort?“ and “Is there enough time to do everything?“), and six items for supervisor support and co-worker support (e.g., “Do your co-workers help you?“ and “Do others understand if you have a bad day?“), with four possible answers for each item (1: never; 2: rarely; 3: sometimes; 4: often). The job demands, job control, and social support items were added together and the continuous measure was used in the further statistical analysis: the higher the score, the higher the job demands and the lower the job control and social support at work. This questionnaire also includes questions on night work and physical work (e.g., lifting, pushing, and transporting). Physical demands were assessed via five items of the Job Content Questionnaire (e.g., “Does your work require rapid continuous physical activity?” and “Are you required to move or lift very heavy loads in your job?”). Items could be answered on a four-point Likert scale, ranging from 1 (almost never) to 4 (almost always). Good internal reliability was obtained (Cronbach’s alpha: 0.89).

#### 2.5.3. Job Satisfaction

We used the Andrew and Withey job satisfaction questionnaire for job satisfaction. This unidimensional questionnaire measures global job satisfaction [29] and includes five items, with responses given on a seven-point Likert scale ranging from delighted (1) to terrible (7). Job satisfaction demonstrated a reliability coefficient of 0.89.

### 2.6. Internal Resources

SRH is a multifunctional measure that this study uses as an indicator of internal resources [18]. SRH is described by the first and the second questions from the SF-36 Health Survey, a self-report questionnaire in which a generic outcome measure is designed to examine self-perceived health status [30]. The first question aims to investigate how the respondent perceives their current health status (“In general, would you say your health is excellent, very good, good, fair, or poor?”) and the second question asks respondents to compare their health to their health a year earlier (“Compared to one year ago, how would you rate your health in general now?” (“Much better now than one year ago”, “Somewhat better now than one year ago”, “About the same”, “Somewhat worse now than one year ago”, “Much worse now than one year ago”)).

The participants were also asked if they had experienced any family crises during the previous year (possible answers: “no, not during the last year,” “yes, unemployment,” “yes, divorce,” “yes, incurable disease or death of a close family number,” “yes, serious financial difficulties in the family.”).

### 2.7. Lifestyle

Lifestyle risk factors were also measured. Smoking was indicated by the following responses: “I do not smoke”, “I smoke every day,” “I smoke occasionally,” “I used to smoke, but quit 1–2 years ago,” “I used to smoke, but quit 3–5 years ago,” “I started smoking this year.” If participants answered, “current smoking,” they were subsequently asked the following question: “how many cigarettes did you smoke per day in the past month?” Individuals who smoked at least 20 cigarettes daily were defined as “heavy smokers”.

Alcohol consumption was evaluated by the following question: “How often do you consume alcoholic beverages?” Five answers were possible: “I do not drink at all,” “I drink 2–3 times a year,” “I drink occasionally,” “I drink each month,” “I drink once a week or more frequently,” and “I drink daily,” with higher scores indicating higher levels of alcohol consumption.

Physical activity was evaluated by the following question: “Do you often exercise (e.g., play sports, or run) in a manner that speeds up your breathing, increases your heart rate and causes you to start sweating in your free time for at least 30 min?” Seven answers were possible: “every day,” “4–6 times a week,” “2–3 times a week,” “once a week,” “2–3 times a month,” “a few times a year or less,” and “I cannot exercise due to illness.” Participants who met the criteria of exercising more than five times a week for at least 30 min were considered regular exercisers. The literature shows that this single-item measure is a valid screening tool for determining whether respondents are sufficiently active to benefit their health [31].

Body mass index (BMI) was calculated (weight/height^2^) based on self-reported weight and height. (Normal weight = 18.5–24.9; overweight = 25–29.9; obese ≥ 30). According to one study, self-reported anthropometric measurements of young adults can be used to calculate BMI for weight classification purposes [32].

### 2.8. Statistical Analysis

We used SPSS 24.0 (IBM, Armonk, NY, USA) for our statistical analysis. Skewness (the symmetry of distribution) and kurtosis (the homogeneity of a distribution) coefficients were calculated to check all variables for normality. When the values of skewness and kurtosis of all study variables are in the range of 2 to −2, the distributions of all variables do not significantly differ from the normal distribution (Table S1). As such, linear regression analysis, which requires normality assumption, can be used [33]. Pearson correlations were calculated for the study variables. First, simple linear regression analysis was used for each burnout component (emotional exhaustion, depersonalization, and reduced personal accomplishment) and each predictor separately to check for significance and effect sizes (expressed as regression coefficients). Then, hierarchical linear regression analyses were performed with three blocks of predictors for dependent variables (emotional exhaustion, depersonalization, and reduced personal accomplishment). The first block included the strongest predictors in univariate analyses (job demands, negative acts at work, witnessing bullying during the previous months, bullying exposure duration, bullying effects on workplace and family relations), and the second block included job satisfaction, years in the respondent’s previous job, job control, social support at work, family crises, and demographic factors (age and gender). Education, lifestyle (physical activity, smoking, alcohol, BMI), night work, and physical load at work were added to the third block.

To illustrate the change in effect sizes when controlling for various blocks of variables, we reported regression coefficients (standardized β). The explained variance was evaluated by R-squared. Statistical significance was set at *p* > 0.05.

## 3. Results

We calculated Pearson correlations for the study variables (Table 1). Emotional exhaustion correlated significantly with all study variables, demonstrating the highest correlation with job demands (0.716, *p* < 0.001), meaning higher job demands correspond to higher levels of emotional exhaustion. Emotional exhaustion was also correlated with negative acts (0.503, *p* < 0.001), witnessing bullying during the previous six months (0.654, *p* < 0.001), bullying affecting workplace and family relations (0.555, *p* < 0.001), and job satisfaction (0.251, *p* < 0.001). Negative correlations were also found with job control (−0.179, *p* < 0.001) and social support at work (−0.293, *p* < 0.001), indicating that higher job control and social support at work lead to lower levels of emotional exhaustion.

Depersonalization correlated significantly positively with job demands, witnessing bullying during the previous six months, bullying exposure duration, bullying’s effect on workplace and family relations, and job satisfaction, and significantly negatively with job control and social support at work. Reduced personal accomplishment positively correlated with job control and social support at work and negatively correlated with job demands, negative acts, witnessing bullying during the previous six months, bullying exposure duration, and job satisfaction.

Our hierarchical linear regression analysis included three blocks of predictors for the dependent variables (emotional exhaustion, depersonalization, and reduced personal accomplishment) (Table 2).

Table 2 shows the strength of adjusted associations from hierarchical linear regression analyses between the covariates and the dependent variables (emotional exhaustion, depersonalization, and reduced personal accomplishment) described in terms of effect sizes (standardized β) and explained variance (R square). The predictors explained 83.2% of emotional exhaustion, 85.0% of depersonalization, and 77.0% of reduced personal accomplishment.

Job demand was the strongest predictor of emotional exhaustion (standardized β = 0.716, *p* < 0.001) and accounted for 51.3% of the variance in the simple regression analysis. In the final model, the effect size of job demands decreased but remained large (standardized β = 0.345) and statistically significant (*p* < 0.001). The effect size of witnessing bullying during the previous six months also diminished (standardized β = 0.654, *p* < 0.001 in the simple regression analysis and standardized β = 0.297, *p* < 0.001 in the final model). Witnessing bullying and job control were the strongest predictors of depersonalization. Negative acts were weaker predictors with lower effect sizes for emotional exhaustion and depersonalization. However, the indicator “negative acts at work” was the strongest predictor of reduced personal accomplishment, followed by job control and social support at work. Physical work (lifting, pushing, carrying, transporting) was a significant predictor of reduced personal accomplishment (standardized β = 0.381, *p* < 0.001).

Bullying statistics indicate that 28.3% (240 cases) of bullying experiences came from superiors, 17.7% (150 cases) from colleagues, 4.6% (39 cases) came from subordinates, and 2.1% (18 cases) from clients. In our study, the most prevalent negative acts were the following: “Someone withholding information which affects your performance”; “Being exposed to an unmanageable workload”; “Being the subject of excessive teasing and sarcasm”; “Being humiliated or ridiculed in connection with your work”; “Being ordered to do work below your level of competence”; “Pressure not to claim something which by right you are entitled to (e.g., sick leave, holiday entitlement, or travel expenses)”; “Being given tasks with unreasonable or impossible targets or deadlines”; “Being ordered to do work below your level of competence”.

Education (university level) was a significant predictor of not only all three burnout components (emotional exhaustion, depersonalization, and reduced personal accomplishment) but also SRH, physical activity, and BMI. Family crises, smoking, alcohol, BMI, and physical inactivity were significant predictors of reduced personal accomplishment.

## 4. Discussion

This study has investigated the impacts of psychosocial factors at work, sociodemographic factors, and lifestyle factors on the three dimensions of burnout (emotional exhaustion, depersonalization, and reduced personal accomplishment). We used hierarchical regression analysis to reveal the most important burnout predictors based on a representative sample of telecommunication sales managers in Lithuania. Our results indicate that job demands most strongly predicted emotional exhaustion, with witnessing bullying at the workplace the second most substantial predictor in terms of effect size. Our results align with those of many other studies concerning the effects of high job demands [34], job tension [9], or work overload [6,35] on emotional exhaustion. A systematic review of scientific articles concerning psychologists treating burnout demonstrated that a heavy workload relates positively to the burnout experienced by psychologists themselves, with that burnout detrimentally affecting not only individuals but also the people receiving their care [36].

The results of investigating bullying were rather astonishing, with witnessing bullying at the workplace a substantial predictor of emotional exhaustion among telecommunication sales managers This result aligns with that of a study of teachers that also confirmed that witnessing bullying may be associated with emotional exhaustion [17]. Elsewhere, a longitudinal study of workers in large industrial enterprises concluded that the overwhelming feelings of stress can impact not only the target of the bullying behavior but also bystanders to the bullying [37]. Workplace bullies (that is, people who belittle, humiliate, and threaten their co-workers), cost organizations billions of dollars each year [38]. Notably, witnessing bullying was also the strongest predictor of depersonalization, further confirming the seriousness of the process of triadic interaction enacted by the social roles of bully, victim, and bystander [37]. Interestingly, Barling’s discussion of primary and secondary victims of workplace violence suggests that secondary victims are employees who were not themselves victims but whose observations, fears, and expectations have changed due to being exposed to violence [39]. Consequently, bystanders to bullying could be considered secondary targets. That is, in bullying-exposed work environments, bystanders are more likely to display symptoms of depression than non-exposed employees [37]. Different investigations have suggested that bullying not only negatively affects the productivity of targets but also adversely affects bystanders to bullying behavior [40]. Furthermore, bystanders more often leave their jobs due to their contact with bullying than non-exposed workers [41]. In our study, a direct bullying experience as a negative act at work was the strongest predictor of reduced personal accomplishment among telecommunication sales managers (standardized β = 0.518, *p* < 0.001). The finding that 28.3% of bullying experiences came from superiors, 17.7% from colleagues, 4.6% came from subordinates, and only 2.1% from clients confirms that the most important source of bullying in the working environment among telecommunication sales managers is superiors. Cross-sectional data from a sample of 2742 service workers confirmed that emotional demands from both sources (clients and colleagues) were associated with higher levels of emotional exhaustion [42].

Our study observed not only job control but also social support at work to be the strongest predictors of reduced personal accomplishment, suggesting that these psychosocial factors at work might trigger reduced personal accomplishment among telecommunication sales managers. Lewin [1], in his study on burnout among salespeople, argued that the burnout process among salespeople begins as a result of weakening job performance, which leads to self-perceptions of diminished accomplishment. As perceptions of reduced personal accomplishment increase, salespeople begin to feel emotionally exhausted. They also tend to distance themselves from some or all of their clients, superiors, and organization, depersonalizing their interactions with those they view as contributors to their diminished state. The depersonalization of client, superior, and organizational relationships increases feelings of emotional exhaustion. Therefore, our study investigating the three dimensions of burnout adds to the scientific literature concerning the possible mechanism driving the process of burnout. It should be a priority of longitudinal studies to confirm or reject this possible motion [1].

Furthermore, our study revealed that job control predicted depersonalization and, to a lesser extent, emotional exhaustion, a finding that coincides with a previous study addressing middle-aged employees [43]. Meanwhile, the findings concerning bullying from superiors and the lack of social support correspond to previous findings suggesting that the lack of time and support from superior staff contributed to the development of burnout among nursing managers [44], aggressive and non-supportive supervision contributed to burnout [9], and positive sales manager support significantly and directly negatively affected the emotional exhaustion of salespersons [1]. Elsewhere, one study showed that the more support received from an organization, the less burnout that was experienced [45], and another demonstrated that support from superiors represents a buffer between job-related stress and stress-related effects [10].

We investigated other job-related predictors of burnout among telecommunication sales managers and found that the length of employment in the company, night work, and job satisfaction were associated with all three burnout dimensions. Studies have shown that job dissatisfaction when starting a job negatively impacts worker motivation and stimulates feelings of reduced personal accomplishment, depersonalization, and emotional exhaustion [11]. Night work (in addition to day work) was the strongest predictor of emotional exhaustion and (to a lesser extent) depersonalization and reduced personal accomplishments. A study considering nurses indicates that night work has been associated with changes in biological functions that contribute to physical and mental disorders [46], especially because nurses often address factors that generate occupational stress at night, which potentially affects their mental health [47]. The finding concerning the length of employment at the company coincides with an investigation of physicians that concluded that the longer the length of service, the higher the level of stress and the higher the risk of burnout [23]. It is apparent that employees cannot adjust to increasing occupational stress over the course of their careers. Meanwhile, we found that physical work is related to personal accomplishment and (to a lesser extent) inversely to depersonalization, serving as a protective mechanism, potentially because physical load contributes to venting negative feelings, improving an individual’s general mental health.

Higher education was also associated with all three burnout dimensions, indicating that persons with a university education have more job responsibilities and duties. This correlates with the findings of Yilmaz (2018), who observed a correlation between educational level and burnout [48]. Women were more prone to all burnout dimensions in our study, with gender representing an important sociodemographic variable that has, nonetheless, been inconsistently correlated with burnout. For example, one study reported that being male was associated with experiencing higher levels of burnout [49]; however, another study reported that gender was a predictor of emotional exhaustion and personal accomplishment, with higher burnout levels among females [50]. We found no consistent associations between age and burnout dimensions, probably because our sample was quite young overall (mean age 27.78 y ± 6.69 [SD]), and the total length of service was only 6.13 y ± 5.58 (SD). It is worth mentioning that the occupation of sales managers in Lithuania is objectively “young” because there were no sales managers in the country until around 25 years ago. Family crises predicted reduced personal accomplishment in our study, suggesting that everyday stress increases the general stress response, leading to stress-related outcomes [24].

Although the concept of SRH has been used in different ways by various investigations, we considered the holistic approach of its correspondence to internal resources, such as optimism, vigor, and perceived control, which are responsible for better managing stress-induced reactions [18]. In our study, SRH predicted all three burnout dimensions, meaning that persons who rate their health low more frequently experience signs of depersonalization, emotional exhaustion, and reduced personal accomplishment. Notably, another representative survey also revealed a substantial correlation between SRH and burnout [51].

Finally, we observed consistent associations between lifestyle factors and burnout dimensions. For example, BMI was a significant predictor of reduced personal accomplishment. A recent systematic review suggested that physical activity constitutes an effective medium for reducing burnout [52], and a study concerning the associations between burnout and health behaviors concluded that burnout contributes to excessive social drinking among ambulance workers [53]. Meanwhile, comparing burnout scores between smokers and non-smokers in a study of mental health professionals indicated that smoking was related to higher levels of emotional exhaustion, depersonalization, and reduced personal accomplishment [54].

In conclusion, our study of a representative sample of telecommunication sales managers in Lithuania demonstrated that burnout is a complex phenomenon with a multifactorial origin. Our study confirms the hypothesis that various psychosocial factors at work, sociodemographic factors, and lifestyle factors contribute to burnout. Job demands and witnessing bullying at the workplace represented the most powerful predictors of emotional exhaustion, followed by SRH, night work, higher education, and physical inactivity. Witnessing bullying at the workplace, job control, SRH, and physical inactivity were the strongest predictors of depersonalization. Finally, direct experience of negative acts at the workplace, job control, social support at work, bullying exposure duration, family crises, physical inactivity, smoking, alcohol use, and BMI were the most important predictors of reduced personal accomplishment. Notably, physical work served as a protective mechanism for reduced personal accomplishment. Although higher education, being female, job satisfaction, and length of time at the company were weaker predictors of all three burnout dimensions, they demonstrated significant effects.

### Strengths and Limitations

The representative sample of telecommunication sales managers, the high response rate, and the concomitant investigation of many burnout predictors represent the strengths of our study. Meanwhile, the limitations include self-reporting and a cross-sectional study design that precluded us from making causal inferences about the impact of the investigated predictors on outcomes. Although a longitudinal study of burnout development would be preferable, such designs are expensive, demanding, and difficult to organize, especially in the context of a volatile population (i.e., telecommunication sales managers). A longitudinal design would permit the examination of the reciprocal and bidirectional associations between burnout dimensions and the associated variables. Nonetheless, our study has confirmed the need to investigate the predictors of all three burnout dimensions, and future research on burnout should be organized around this finding.

## 5. Conclusions

The present study has filled the gap in the literature concerning a simultaneous approach to all three burnout dimensions. It reveals that high job demands, low job control, and low social support at work are associated with burnout, and contributes to the novel understanding that bullying plays an integral role in burnout. Direct negative acts experienced in the workplace are associated with reduced personal accomplishment while witnessing bullying towards co-workers is associated with emotional exhaustion and depersonalization. This finding provides insight to guide burnout prevention programs in occupational health settings, which might consider introducing bullying prevention measures in workplaces. This problem should also be addressed by legislation and executive documents.

Occupational health professionals should advise employees to follow the guidelines on lifestyle measures that can improve physical activity levels, mitigate harmful behavioral habits, and reduce BMI because these factors have deep associations with various burnout dimensions. Furthermore, improving the psychosocial situation in the workplace can be achieved by increasing the support that employees receive from superiors and co-workers and attempting to provide more freedom at work in the sense of decision latitude and skill discretion. These would be steps toward resolving the problem of burnout in the working environment, which has yet to be solved despite the many preventive programs already implemented.

## Figures and Tables

**Table 1:** Means, standard deviations, and correlations of study variables.

	Mean ± SD	1	2	3	4	5	6	7	8	9	10	11
1. Emotional exhaustion	18.18 ± 10.82	1										
2. Depersonalization	14.91 ± 8.42	0.879 **	1									
3. RPA	17.66 ± 8.10	−0.510 **	−0.552 **	1								
4. Job demands	12.09 ± 2.47	0.716 **	0.545 **	−0.306 **	1							
5. Job control	13.99 ± 3.69	−0.179 **	−0.254 **	0.399 **	−0.437 **	1						
6. Social support	14.41 ± 4.63	−0.293 **	−0.175 **	0.435 **	−0.484 **	0.689 **	1					
7. Negative acts	42.52 ± 17.09	0.503 **	0.624 **	−0.271 **	0.151 **	−0.276 **	−0.288 **	1				
8. Witnessing bullying	1.69 ± 0.89	0.654 **	0.718 **	−0.123 **	0.369 **	−0.008	−0.153 **	0.609 **	1			
9. BED	1.98 ± 1.45	0.447 **	0.586 **	−0.148 **	0.324 **	−0.132 **	−0.052	0.522 **	0.624 **	1		
10. BEWFR	1.84 ± 1.12	0.555 **	0.588 **	−0.008	0.440 **	−0.134 **	−0.239 **	0.356 **	0.702 **	0.739 **	1	
11. Job satisfaction	24.72 ± 5.38	0.251 **	0.403 *	−0.123 **	0.161 **	−0.105 **	−0.075 *	0.292 **	0.277 **	0.288 **	0.247 **	1

Notes. * p < 0. 05; ** *p* < 0.001; SD: standard deviation; RPA: reduced personal accomplishment; BED: bullying exposure duration; BEWFR: bullying’s effect on workplace and family relations.

**Table 2:** Predictors of components of burnout.

	Emotional Exhaustion		Depersonalization		Reduced Personal Accomplishment	
	Standardized β	*p*	Standardized β	*p*	Standardized β	*p*
Job demands	0.345	<0.001	0.051	0.093	−0.070	0.063
Negative acts at work	0.121	0.04	0.184	<0.001	−0.518	<0.001
Witnessing bullying	0.297	<0.001	0.374	<0.001	−0.087	0.067
BED	0.085	0.083	0.102	0.028	−0.214	<0.001
BEWFR	0.198	<0.001	0.192	<0.001	−0.011	0.822
Job Control	−0.081	0.009	−0.304	<0.001	0.468	<0.001
Social support at work	−0.086	0.016	−0.034	0.309	0.342	<0.001
Job satisfaction	0.071	<0.001	0.183	<0.001	−0.039	0.097
LE	0.187	<0.001	0.184	<0.001	−0.156	<0.001
Night work	0.227	<0.001	0.204	<0.001	−0.190	<0.001
PW	0.002	0.943	−0.114	<0.001	0.381	<0.001
Gender	0.052	0.011	0.132	<0.001	−0.104	<0.001
Age	−0.104	<0.001	−0.003	0.922	0.030	0.350
Education	−0.207	<0.001	−0.117	<0.001	0.179	<0.001
Physical inactivity	0.214	<0.001	0.298	<0.001	−0.287	<0.001
Smoking	0.008	0.754	0.056	0.018	−0.294	<0.001
Alcohol	0.157	<0.001	0.096	<0.001	−0.223	<0.001
Body mass index	0.098	<0.001	0.093	<0.001	−0.297	<0.001
Family crises	0.046	0.082	0.021	0.402	−0.418	<0.001
Self-rated health	0.268	<0.001	0.304	<0.001	−0.127	<0.001
R square	0.832	<0.001	0.850	<0.001	0.770	<0.001

Notes. SD: standard deviation; BED: bullying exposure duration; BEWFR: bullying’s effect on workplace and family relations; LE: length of employment at the company; PW: physical work (e.g., lifting, pushing, carrying, and transporting).

## Data Availability

The datasets collected and analyzed during the current study are available from the corresponding author upon reasonable request.

## Supplementary Materials

The following supporting information can be downloaded at: https://www.mdpi.com/article/10.3390/ijerph191811249/s1.
