# Supplementary material for: Burnout among Telecommunication Sales Managers
Source: Int J Environ Res Public Health. 2022 Sep 7;19(18):11249. doi: 10.3390/ijerph191811249 (PMC9517578; doi:10.3390/ijerph191811249)
Supplement: Supplementary file 1 [file ijerph-19-11249-s001.zip › ijerph-1863409-supplementary.pdf]

Table S1. Means, standard deviations (SD), and normality tests of the study variables

	N	Mean	SD	Skewness		Kurtosis	
				Statistic	Std. Error	Statistic	Std. Error
Emotional exhaustion	849	18.18	10.82	.181	.084	-1.081	.168
Depersonalisation	849	14.91	8.42	.329	.084	-.628	.168
RPA	849	17.66	8.10	-.351	.084	-.315	.168
Job demands	849	12.09	2.47	.023	.084	-.520	.168
Job control	849	14.00	3.69	-.221	.084	-.681	.168
Social support at work	849	14.41	4.63	.467	.084	-.891	.168
Negative acts at work	849	42.52	17.09	.294	.084	-1.413	.168
Bullying exposure duration	849	1.98	1.44	1.675	.084	1.870	.168
Bullying witnessing	849	1.69	0.89	1.011	.084	-.109	.168
BEWFR	849	1.84	1.12	.909	.084	-.746	.168
Job satisfaction	849	24.72	5.38	.416	.084	.179	.168
LE	849	3.03	3.17	1.582	.084	1.859	.168
Night work	849	3.22	0.84	-.440	.084	-1.455	.168
PW	849	3.20	0.83	-.569	.084	-.819	.168
Gender	849	1.62	0.49	-.504	.084	-1.750	.168
Age	849	27.78	6.69	1.383	.084	1.064	.168
Education	849	1.42	0.75	1.394	.084	.223	.168
Physical activity	849	4.12	1.71	-.231	.084	-.854	.168
Smoking	849	2.44	1.41	.939	.084	.019	.168
Alcohol	849	2.94	1.66	.124	.084	-1.408	.168
Body mass index	849	23.58	2.70	-.052	.084	-.248	.168
Family crises	849	1.47	0.89	1.639	.084	1.241	.168
Self-rated health	849	2.28	0.83	.110	.084	-.593	.168

Notes. SD–standard deviation. Std. Error–standard error. RPA–Reduced personal accomplishment. BEWFR–Bullying effect on workplace and family relations. LE– Length of employment in the company. PW–Physical work (lifting, pushing, carrying, transporting).
